# Aging affects motor planning for grasping everyday objects: A case control study

**DOI:** 10.1371/journal.pone.0347103

**Published:** 2026-04-20

**Authors:** Guillaume Galliou, Lucette Toussaint, Thomas Rulleau

**Affiliations:** 1 Université de Poitiers, Université de Tours, CNRS, CeRCA, Faculté des Sciences du Sport, Poitiers, France; 2 Nantes Université, CHU Nantes, Direction de la Recherche et de l’innovation, Mouvement - Interactions - Performance, MIP, Nantes, France; University of Giessen: Justus-Liebig-Universitat Giessen, GERMANY

## Abstract

**Background and purpose:**

The ability to anticipate the movement of objects to be grasped is essential for older people to remain independent in their daily lives and to avoid accidents at home. This study was designed to investigate how normal aging affects motor planning for the grasping of everyday objects.

**Method:**

Sixty-nine right-handed adults with no cognitive impairment participated in the study. They were divided according to age into a young group (n = 30, aged 18–30 years) or an old group (n = 39, aged 65 years and older). Their motor-planning abilities were assessed using an object-grasping judgment task, their manual dexterity with a timed peg placement task and their motor imagery abilities with the Vividness of Movement Imagery Questionnaire. For all variables, a nonparametric Mann–Whitney U test was used to identify differences between the two groups. Spearman’s rho correlation was used to examine the relationships between motor-planning ability and manual dexterity score, as well as between motor-planning ability and motor imagery ability, in each group.

**Results and discussion:**

Compared with the young group, the old group took longer to choose the correct grasp during the object-grasping judgment task and had poorer manual dexterity. Analysis of the correlations suggested that the older participants who had the longest response times in the object-grasping judgment task tended to place the fewest pegs in the manual dexterity task and to have the lowest motor imagery skills.

**Conclusions:**

Aging may affect motor planning in a particular way. It does not affect the ability to build the expected motor plan, but appears to slow down the information processing needed to build that plan. This difficulty seems to be linked to the decline in manual dexterity and motor imagery skills as people age.

## Introduction

Aging is a slow, progressive process that has a negative impact on human movement control. In addition to the loss of strength [1], the effect of aging is associated with reduced control of upper limb fine motor skills, gait and balance [2,3]. In their review [4], Carmeli et al. reported that the functional ability of the hand appears to be preserved up to the age of 65 years and then gradually declines. This reduced control over hand movements can affect older people’s ability to perform activities of daily living and have a negative impact on their quality of life. In the present study, we focused specifically on how motor planning for the grasping of everyday objects is affected by normal aging.

Grasping an object to use it effectively requires good motor-planning skills, i.e., the ability to anticipate its use to decide how to grasp it. The effective grasping of an object to facilitate its subsequent use is the basis of the end-state comfort effect (or ESC effect), first described by Rosenbaum 30 years ago [5–7]. The ESC effect emphasizes the tendency to grasp an object differently depending on what one intends to do with it, with a preference for final comfort over initial comfort to later implement the action comfortably and efficiently [7,8]. An everyday example of the final comfort effect can be observed in the task of picking up a spilled glass, described by Rosenbaum [5]. He also describes another task, that of placing a two-colored bar on a disc. The goal being to use the right hand to place the black end of the bar on the left on a disc, individuals will generally position their hand in supination. This allows the wrist to rotate comfortably and stably, without adopting extreme joint angles. However, if the goal is to use the right hand to place the gray end of the bar on the right onto a disc, the hand will be positioned in pronation. In both cases, the initial orientation of the hand reflects the motor planning necessary to reach a final position that allows the desired action to be performed effectively. Respecting the ESC effect is crucial for performing actions efficiently [8]. Planning hand and wrist positions in anticipation of the final posture reduces awkward movements and minimizes the risk of accidents, especially during everyday activities at home.

To date, much of the scientific literature has focused on the development of the ESC effect in grasp planning using laboratory tasks such as bar-transport handle [9–11] or disc rotation tasks [12–14]. These studies highlighted the improvement in motor-planning abilities throughout childhood into adulthood. Others have focused on the development of the ESC effect in more familiar tasks, corresponding to the manipulation of everyday objects in the overturned glass or the spoon tasks [15,16], which maximize planning performance. These studies highlighted that habits or familiarity with objects influence motor planning in children.

While motor-planning abilities improve throughout childhood into adulthood, recent studies, although few, have reported that the ESC effect decreases as people age due to a decline in cognitive ability [17–19]. These findings have been highlighted in studies using bar-transport tasks, horizontal dowel tasks and/or handle rotation tasks. However, when performing a familiar task such as grasping an upright or inverted glass, older adults did not always differ from young adults with respect to sensitivity to end-state comfort [20]. As previously reported for children, some authors have suggested that older adults may perform poorly on laboratory tasks because of their lack of familiarity with the stimuli used [21,22].

In the present study, we investigated the effect of normal aging (young versus old groups) on the ability to anticipate the grasp of everyday objects by examining ESC in the context of rehabilitation. We used an object-grasping judgment task (see Toussaint et al.[23] for a similar procedure) in which participants had to judge whether everyday objects displayed on a computer screen should be grasped from above or below, depending on the action associated with them. This task allowed us to evaluate not only the accuracy of the grip (i.e., with respect to the ESC effect) but also the time required to plan the correct grip. The results obtained when using the computer paradigm to assess the motor planning mechanisms of grasping are strongly correlated with those obtained during actual grasping [24]. Response times should be considered as an indirect indicator of motor planning, incorporating action simulation processes, rather than as a pure measure of motor planning of an actual grasp. In addition to the object-grasping judgment task, we assessed the motor imagery ability and manual dexterity of our young and old participants, as performance on this task has been found to be related to these factors [23].

## Method

This retrospective observational study was based on activity data from two centers in France (a private physiotherapy practice and a retirement home). Data were consulted from February 2, 2024 to July 13, 2024.

### Participants

All participants in this convenience sample were volunteers for the study, which was conducted in accordance with the Declaration of Helsinki and was approved by the local ethics committee (Groupe Nantais d’Ethique dans le Domaine de la Santé – GNEDS, n° 24-16-01-112). Only the principal investigator had access to the complete patient file for routine care. The other authors did not have access to identifying data.

Sixty-nine participants declaring themselves to be right-handed were included in the “young” or “old” group according to their age. In order to limit the cognitive load for the patients and avoid redundant questionnaires, the Edinburgh Laterality Inventory was not used, as laterality was already collected through self-reporting. In the young group, the participants were aged between 18 and 30 years. In the old group, the participants were aged 65 years or older. The characteristics of each group are shown in Table 1. All the participants appeared to have no obvious cognitive impairment, as indicated by their Mini-Mental State Examination scores of 24 or above [25], which classifies their level of cognitive impairment as normal [26,27]. Their motor imagery abilities were assessed using the Vividness of Movement Imagery Questionnaire (VMIQ) [28], in which participants rated their ease/difficulty of motor imagery using a 5-point Likert scale. A score close to 1 indicates good imagery ability (i.e., clear and vivid images), whereas a score close to 5 indicates poorer imagery ability (i.e., unclear and not vivid images). The VMIQ distinguishes between internal (or first-person) and external (or third-person) imagery perspectives. Although motor imagery abilities were relatively good for all participants, the VMIQ scores (Table 1) indicated clear and reasonably vivid imagery capacities (VMIQ scores close to 2), and the Mann‒Whitney tests revealed that imagery scores were significantly better in the young group than in the old group for the first-person perspective (U_67_ = 804.5, p = 0.003), whereas no difference was observed for the third-person perspective (U_67_ = 720.5, p = 0.10).

**Table 1:** Means (standard deviations) of the characteristics of the participants in the young and old groups.

	Young group (n = 30)	Old group (n = 39)
Age (years)	24 (±4)	78 (±10)
Sex	17 females, 13 males	22 females, 17 males
MMSE	30 (±0)	27.1 (±1.9)
VMIQ 1^st^-person	1.79 (±0.67)	2.53 (±1.20)
VMIQ 3^rd^-person	1.86 (±0.62)	2.33 (±1.02)

### Materials, tasks and procedures

All the participants performed two consecutive tasks, the first assessing motor-planning abilities and the second assessing manual dexterity.

### Motor-planning abilities

**Fig 1:**
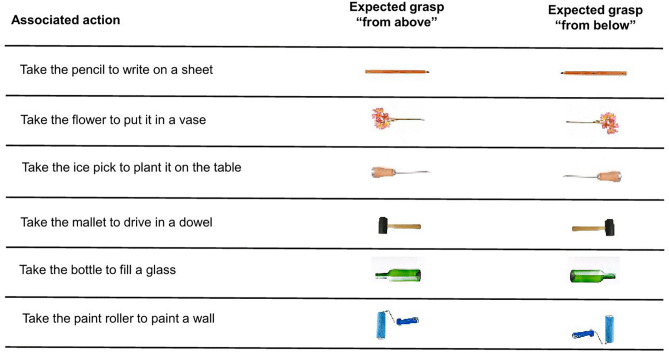
Illustration of the objects used with the expected grasp according to their orientations.

We assessed motor-planning abilities with an object-grasping judgment task (see Toussaint et al.[23] for a similar procedure). The participants were seated 50 cm in front of a computer. Six objects (pencil, flower, ice pick, mallet, bottle and paint roller) were displayed on the screen, with their functional parts either on the left or on the right (Fig 1). Before an object appeared on the screen, a sentence describing its associated action was shown for 3500 ms. For example, the sentence “take the pencil to write on a sheet” was read aloud by the participant before the pencil (lead to the left or right) appeared on the screen and he decided on the appropriate grip. In this task, the participants had to decide as quickly and accurately as possible whether to grasp the object displayed from above or below when using their right hand by pressing the “q” or the “s” key, respectively (QWERTY keyboard). The instruction ‘from above’ means that the participant should grasp the object by the functional part with the hand in pronation, while ‘from below’ means using the hand in supination. A white screen appeared for 500 ms before another trial was presented (i.e., a sentence followed by an object). For all the participants, regardless of their group, we recorded a total of 60 responses, i.e., 5 blocks of 12 randomly presented trials (6 objects × 2 expected grasps). Each session lasted approximately 10 min.

We used the E-Prime 2.0 software package (Psychology Software Tools Inc., Pittsburgh, PA, USA) to show the sentences and objects and to record the accuracy and response times of the participants.

### Manual dexterity

We assessed participants’ manual dexterity using a task in which they had to use their right hand to place small pegs in the holes of a rectangular board. The pegs were 10 mm high and 4 mm in diameter. The board was 33 cm long and 8 cm wide and had 12 rows of 4 holes. The pegs had to be placed in the holes one at a time, and the participants had to place as many pegs as possible in 30 seconds.

### Statistical analysis

For the object-grasping judgment task, we computed accuracy (i.e., the percentage of correct answers) and response times for all trials. Response times were analyzed for correct answers only. Moreover, response time outliers (±2.5 standard deviations) were excluded from the analyses (less than 3.5% in each group). We used the Shapiro‒Wilk test to check whether the variables followed a normal distribution before we carried out the statistical analyses. Because none of the variables followed a normal distribution, we used a nonparametric Mann‒Whitney U test to assess differences in accuracy and response time in the object-grasping judgment task and in the manual dexterity score between the young and old groups. Finally, we used Spearman’s rho correlation to examine the relationships between motor-planning ability and manual dexterity score, as well as between motor-planning ability and motor imagery ability, in each group. All analyses were carried out using JASP® software (version 0.16.1.0), and the alpha threshold was set at 0.05. For the Mann‒Whitney test, the effect size is given by the rank biserial correlation.

All raw results are available at

https://osf.io/2xbev/?view_only=dc58734bcb9d4eaeb3dd714a4445309f

## Results

### Motor-planning abilities

**Fig 2:**
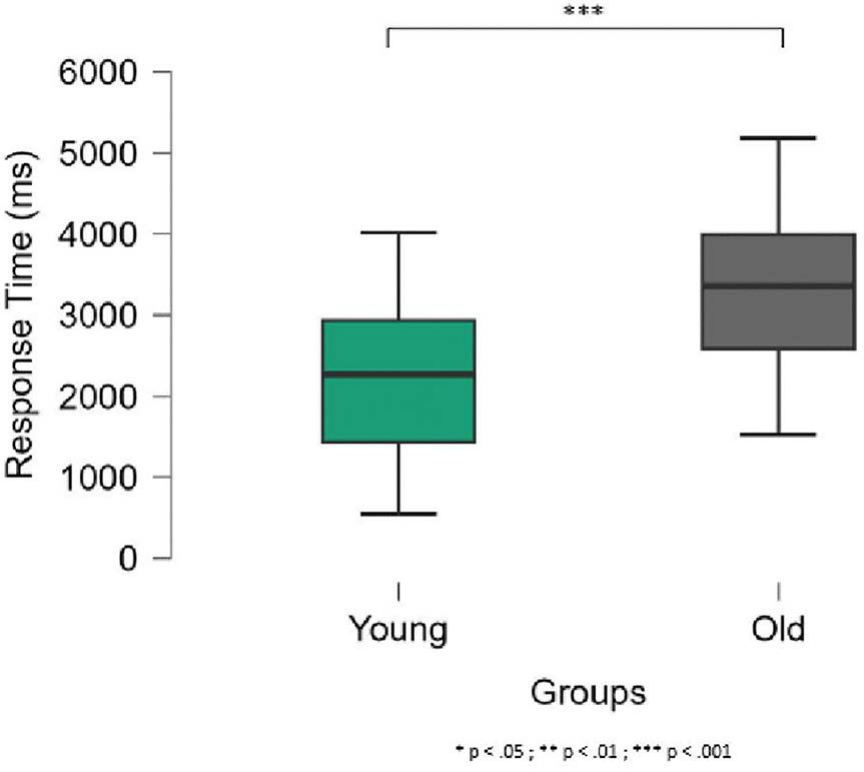
Response times (ms) in the object-grasping judgment task in the young and old groups. The error bars indicate the standard deviation.

The nonparametric Mann‒Whitney U test performed on the accuracy of the object-grasping judgment task revealed no statistically significant differences between young and old participants (U_67_ = 572.0.0, p = .88), with an average mean of 78% (SD = 15%). However, the two groups significantly differed in response times (U_67_ = 860.5, p < .001, effect size = 0.47), with older participants taking 980 ms more than younger participants to determine whether an object has been grasped from above or below, depending on the action to be performed (see Fig 2).

### Manual dexterity

**Fig 3:**
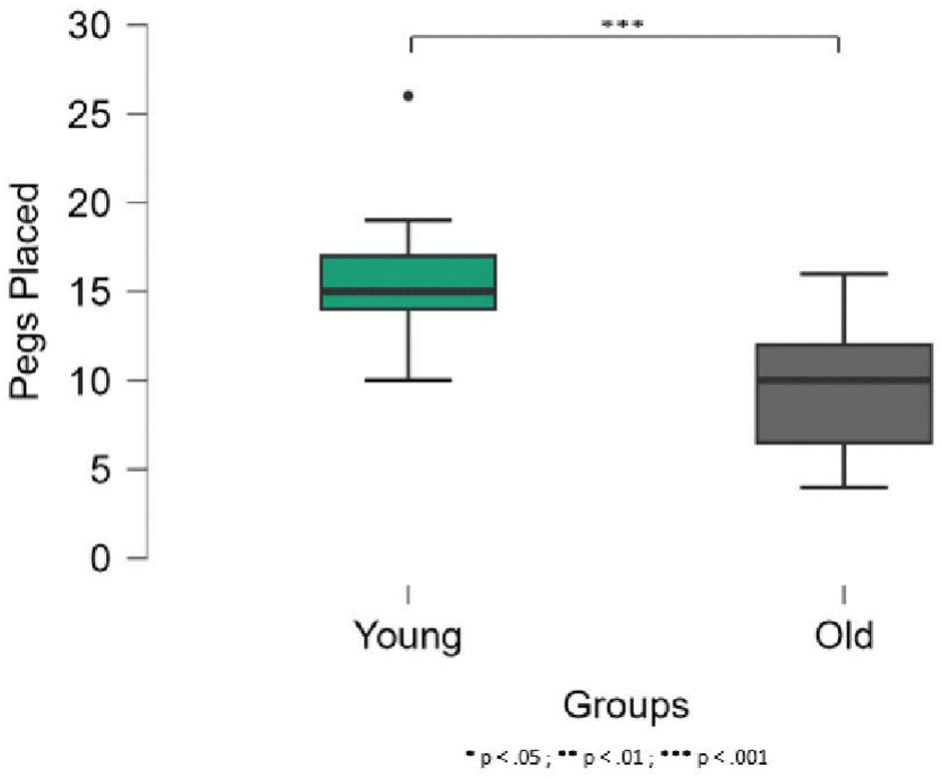
Number of pegs placed in 30 s in the manual dexterity task. The error bars indicate the standard deviation.

The results of the manual dexterity task (i.e., the number of pegs placed on the board in 30 s) differed significantly between the two groups (U_67_ = 44.5, p < .001, effect size = 0.92), with a greater number of pegs placed by the younger participants (mean = 15.4, SD = 2.8) than by the older participants (mean = 8.5, SD = 3.1) (see Fig 3).

### Correlation between motor-planning ability and manual dexterity

**Fig 4:**
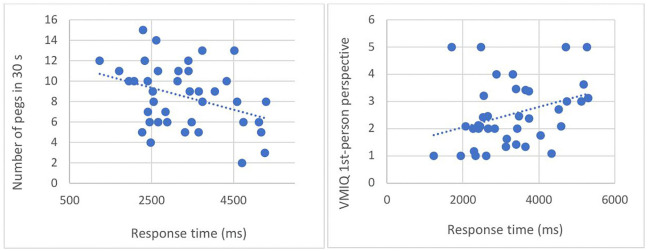
Correlation between response times in the object-grasping judgment task and the number of pegs placed on the board in the manual dexterity task (left fig.) as well as the VMIQ scores from the first-person perspective (right fig.) for the old group. Note, ns: non-significant correlation, * p < .05.

Spearman’s rho correlation between performance on the object-grasping judgment task (accuracy and response time) and manual dexterity scores is reported in Table 2. A significant negative correlation appeared only in the old group (r = −0.33, p = .03). The participants who had longer response times in the object-grasping judgment task were those who placed the fewest pegs in the manual dexterity task (Fig 4, left side).

**Table 2:** Spearman’s rho correlation coefficients between performance on the object-grasping judgment task and scores on the manual dexterity task as well as VMIQ scores.

			VMIQ Scores	
		Manual dexterity	1^st^-person perspective	3^rd^-person perspective
**Object-grasping judgement task**				
**Accuracy (%)**	Young group (n = 30)	0.17 ns	−0.03 ns	−0.02 ns
	Old group (n = 39)	−0.10 ns	−0.01 ns	−0.03 ns
**Response time (ms)**	Young group (n = 30)	0.03 ns	0.33 ns	0.16 ns
	Old group (n = 39)	−0.33*	−0.37*	−0.28 ns

### Correlation between motor-planning ability and motor imagery

As shown in Table 2, only the old group showed a significant positive correlation between response times in the object-grasping judgment task and scores from the first-person perspective of the VMIQ (r = 0.37, p = 0.02). This correlation revealed that the participants who had longer response times in the object-grasping judgment task were those with the lowest motor imagery abilities (Fig 4, right side).

## Discussion

The aim of this study was to test whether aging might affect motor-planning abilities for grasping everyday objects. To this end, we asked young (aged between 18 and 30 years) and old participants (aged 65 years and older) without cognitive disorders (MMSE>24 [26]) to perform an object-grasping judgment task. We also evaluated their motor imagery and manual dexterity. The main results showed that for the same level of accuracy with respect to the ESC (average 78%), the older participants needed more time to program the appropriate grasping plan. In fact, they took 980 ms longer than the younger participants to decide whether an object should be grasped from above or below, depending on the action to be performed with it. The older participants also had poorer motor imagery abilities and less efficient manual dexterity than the younger participants. For the older participants only, we reported significant but modest correlations between response time in the object-grasping judgment task and manual dexterity, as well as between response time in the object-grasping judgment task and motor imagery ability. These correlations are therefore informative but should be interpreted cautiously due to their weak magnitude.

First, these results confirm the detrimental effects of aging on the ability to plan the grasping of everyday objects. However, in contrast to some studies [17–19], aging does not seem to affect the way objects should be grasped according to their use but rather the time it takes to build the appropriate grasping plan. In the present experiment, we effectively found that older adults took longer to complete the action plan for grasping everyday objects than younger adults did while no difference between groups appeared for accuracy (i.e., for the percentage of correct responses). Note that no significant differences in ESC sensitivity between young and old participants were previously reported by Sharoun et al.[20] when participants were asked to grasp or mimic grasping a familiar object (i.e., an upright or inverted glass). On the basis of these findings, we suggest that aging affects motor planning in a particular way depending on the familiarity of the objects to be grasped (see also Bock & Steinberg [22] and Cicerale et al.[21] for a discussion of poorer performance on laboratory tasks with unfamiliar stimuli). Therefore, we can assume that older adults retain the ability to perform everyday grasping but need more time to perform efficiently.

How can the detrimental effects of aging on motor planning be explained? Some studies have highlighted the decline in cognitive abilities such as executive function [17–19,29]. Although a more comprehensive assessment of cognitive deficits would have been necessary, the Mini-Mental State Examination (MMSE) scores obtained by the participants in our study suggest preserved cognitive abilities. Their MMSE scores were of 24 or above, which classify their level of cognitive impairment as normal [26]. However, it should be noted that their mean MMSE score (27.1) was lower than that of the young group [30]. Therefore, our study does not challenge the hypothesis that a decline in cognitive ability may be the cause of less efficient object-grasping planning. However, as developed below, by assessing motor imagery and manual dexterity, we are able to refine our understanding of the factors behind less efficient motor planning with advancing age.

Motor imagery is a key factor in motor planning. In ESC tasks, participants must effectively anticipate their future bodily states to achieve the intended action goal. This means that if a participant has a lower motor imagery ability than another participant does, his or her ESC performance should be worse. The relationship between imagery ability and ESC performance has been highlighted in children [23,30] and young adults but not yet in older adults. The present study added specific information on the importance of motor imagery for ESC in older adults. First, although motor imagery abilities were relatively good in all participants (see Table 1), the first-person perspective imagery score indicated lower imagery abilities for older participants. Second, the participants in the old group with the longest response times in the object-grasping judgment task were those with the lowest reported motor imagery abilities. These results highlight the contribution of motor imagery to planning efficiency, while acknowledging that the observed correlations are modest and should be interpreted cautiously. In addition to highlighting the importance of motor imagery for grasping everyday objects in older volunteers, the present study suggests that the VMIQ, which is easy to administer in clinical settings, is a good tool for gaining insight into potential declines in motor-planning ability in older people.

In addition to the decline in motor imagery ability, differences in motor planning with aging may also be partially explained by physical factors, such as the reduction in wrist range of motion [31] and manual dexterity experienced with aging [18]. These findings have been demonstrated using laboratory tasks (i.e., with unfamiliar stimuli). Our study confirms the relationship between motor planning and manual dexterity in older adults when familiar objects are used. We report a significant correlation between the time required to plan the grasp of everyday objects and manual dexterity in older participants. The participants who had longer response times in the object-grasping judgment task were those who placed the fewest pegs in the manual dexterity task. Given the weak magnitude of these correlations, these results suggest trends rather than strong effects. However, the experimental design of the study does not allow us to determine whether motor-planning impairments are the cause or the consequence of age-related motor impairments. Therefore, the question of whether rehabilitation programs aimed at maintaining high motor-planning skills in older adults should include fine motor interventions remains open and needs to be addressed to protect seniors from potential home accidents.

### Limits

This case-control study compares two populations of different ages. It therefore includes confounding factors in the comparison of the two populations, but the results confirm those previously reported by several authors [18,19].

## Conclusion

This study highlighted the negative effect of aging on motor-planning abilities for everyday objects. Older participants are still able to construct the appropriate plan, but this construction takes longer. This longer time is associated with poorer motor imagery abilities and less efficient manual dexterity, suggesting that both motor and cognitive factors may be essential for successful motor planning. Although further studies are needed to clarify how to optimize geriatric rehabilitation, specifically for action-planning skills, the present study provides a complementary argument to studies highlighting the great impact of combined cognitive and physical training on cognitive function [32,33]. Furthermore, for efficient interaction with everyday objects in the environment, combined training can focus on motor imagery and manual dexterity training. Further studies are needed to clarify which rehabilitation techniques can be implemented to maintain or reactivate motor-planning skills in older patients.
